# High Risk of Herpes Zoster among Patients with Advance Acute Kidney Injury – A Population-Based Study

**DOI:** 10.1038/srep13747

**Published:** 2015-09-03

**Authors:** Wei-Shun Yang, Fu-Chang Hu, Meng-Kan Chen, Wen-Je Ko, Likwang Chen, Kwan-Dun Wu, Vin-Cent Wu

**Affiliations:** 1 Department of Internal Medicine, National Taiwan University Hospital, Hsin-Chu Branch, Hsin-Chu city, Taiwan; 2 College of Medicine, National Taiwan University, Taipei, Taiwan; 3 International-Harvard Statistical Consulting Company, Taipei, Taiwan; 4 Division of Nephrology, Department of Internal Medicine, National Taiwan University Hospital, Taipei, Taiwan; 5 Department of Family Medicine, National Taiwan University Hospital, Hsin-Chu Branch, Hsin-Chu, Taiwan; 6 Department of Surgery, National Taiwan University Hospital, Taipei, Taiwan; 7 Institute of Population Health Sciences, National Health Research Institutes, Zhunan, Taiwan

## Abstract

The risk for herpes zoster (HZ) in acute kidney injury (AKI) survivors was never explored. We identified 2,387 adults in the Taiwan National Health Insurance Research Database who recovered from dialysis-requiring AKI and matched them with non-recovery and non-AKI patients by propensity score. During a mean follow-up of 2.7 years, the incidences of HZ were 6.9, 8.2 and 4.8 episodes per 1,000 person-years in AKI-non-recovery, AKI-recovery and non-AKI group, respectively. The recovery group was more likely to develop herpes zoster than those without acute kidney injury [incidence-rate ratios 1.71, 95% confidence interval 1.16–2.52; p = 0.007]. Patients without acute kidney injury were less likely to develop herpes zoster than those AKI, recovered from dialysis or not (hazard ratio HR 0.66, 95% CI 0.46–0.95). Dialysis-requiring acute kidney injury poses a long-term risk of herpes zoster after hospital discharge. Even patients who have recovered from dialysis still carry a significantly higher risk of developing herpes zoster.

As the collaborative campaign (World Kidney Day, 2013) promoted by ISN (International Society of Nephrology) and IFKF (International Federation of Kidney Foundations) indicated, acute kidney injury (AKI) is now a growing global health alert. It is estimated to occur in 3–5% of the community population annually, and the rate is even higher in hospitalized (5–25%) or ICU patients (30–60%), causing great morbidity/mortality and medical expenses^1^,^2^,^3^,^4^. Even for those AKI survivors with complete recovery of renal function, an elevated risk remains for subsequent chronic kidney disease (CKD), end-stage renal disease (ESRD) and mortality^5^,^6^,^7^. Moreover, distinct elements of the innate and adaptive immune response are also involved in the expression of tissue injury following acute kidney injury. Renal ischemic-reperfusion injury amplifies the humoral immune response to heterologous antigens^8^,^9^. The uremic toxins accumulated during AKI can cause epigenetic modification and presumably account for the increased risk for cancer, sepsis and tuberculosis in AKI survivors shown in large scaled population-based studies^10^,^11^,^12^,^13^. It is therefore reasonable to speculate that AKI, irrespective of the recovery from dialysis, may also pose an impact on other disorders associated with immune derangement.

Herpes zoster (HZ) is the reactivation of latent varicella-zoster virus infection in the nerve ganglia after primary infection, usually when the host’s cellular immunity declines. Herpes zoster and its consequence of post-herpetic neuralgia (PHN) can significantly compromise patients’ life quality and cause medical economic burden^14^. Patients with old age, systemic diseases (e.g. diabetes mellitus), immunosuppressant use or malignancies are at higher risk of HZ reactivation^15^,^16^,^17^. Patients with renal failure are also immunocompromised and not surprisingly at greater risk of HZ reactivation, including not only those with maintenance hemodialysis, peritoneal dialysis or renal transplant^18^,^19^ but also patients with chronic kidney disease (CKD) not yet receiving renal replacement therapy^20^. These findings raise the concern that HZ reactivation is a common burden to patients across the full spectrum of renal impairment. However, the risk of HZ reactivation in patients recovering from AKI is not yet known. We therefore conducted a nationwide observational study to explore the relationship between severe, dialysis-requiring AKI and subsequent HZ reactivation.

## Results

### Patient characteristics

We identified 2,387 patients recovered from severe, dialysis-requiring AKI and matched them with the same number of non-recovery (dialysis-dependent) and non-AKI patients in the Taiwan National Health Insurance database by propensity score matching. Baseline demography, Charlson morbidity index and index hospitalization conditions and transplant (solid organ other than kidney or hematopoietic) were identical in all groups after the matching process. There were slightly more patients with pre-existing liver disease in the control group (p = 0.043) (Table 1), therefore we treated this difference in further analysis with special concern. As for further risks of HZ after the index hospitalization that might also influence our study endpoint, we surveyed the prescription of immunosuppressant agents at discharge and listed steroids and the five most frequently used in Table 1. As for renal function change after index hospitalization, all non-recovery group patients went on receiving long-term renal replacement therapy. Despite that all recovery group patients were able to wean off dialysis at discharge, 20% percent of the recovery group entered CKD stage (ICD-9-CM 585) and 28.3% ended up with ESRD (with catastrophic illness certification) sometime during follow-up after the index hospitalization, contrasting 10.4% and 2.5% in non-AKI group. However, the LHID database does not contain laboratory data, hence the exact renal function change, such as serum creatinine level or estimated glomerular filtration rate change, was not available.

### Endpoint

During a mean follow-up of 2.7 years, the incidences of HZ were 6.9, 8.2 and 4.8 episodes per 1,000 person-years in AKI-non-recovery, AKI-recovery and non-AKI group, respectively. The recovery group was more likely to develop herpes zoster than those without AKI [incidence-rate ratios 1.71, 95% confidence interval 1.16–2.52; p = 0.007]. On the other hand, patients without AKI were most unlikely to develop herpes zoster than both the AKI- recovery and non-recovery groups [hazard ratio HR 0.66, 95% CI 0.46–0.95]. In all three groups, HZ took place mostly within 5 years after index hospitalization (82.1%, 84.6% and 90.5% for AKI-non-recovery, AKI-recovery and non-AKI group, respectively). The mean time from index hospitalization to HZ was 2.7, 2.1 and 2.5 years for AKI-non-recovery, AKI-recovery and non-AKI group, respectively. Despite better renal outcome in the AKI-recovery versus non-recovery group (48.3% CKD plus ESRD versus 100% ESRD), the HZ incidence was not lower in the recovery group.

### Predictors of herpes zoster reactivation

**Figure 1:**
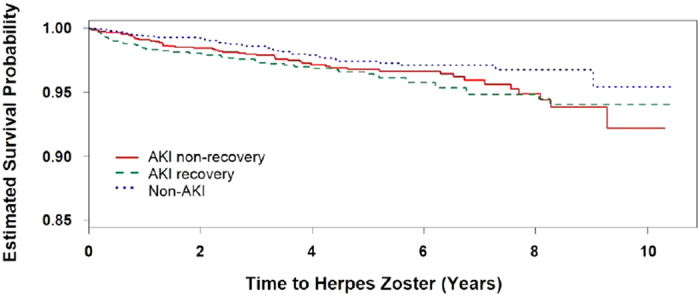
Kaplan-Meier curves of HZ-free survival. *p value for log-rank test 0.0249.

Despite that the pre-discharge conditions were well matched, we observed higher prescription rates of steroids and immunosuppressant agents in AKI non-recovery and recovery groups after discharge. Therefore, to determine whether the higher HZ incidence was confounded by these medications, we performed a Cox proportional hazards model with steroids and immunosuppressant agents as time-dependent covariates (Table 2). As expected, the impact of steroids and immunosuppressant agents was profound on HZ, but AKI survivors (groups 1 and 2 together) still have a HR of 1.54 of getting HZ independently of these medications. Figure 1 shows the stratified Kaplan-Meier curves of HZ-free survival of the three groups.

## Discussion

Aside from the known increased risk of HZ in CKD and ESRD population, we are able to demonstrate for the first time that AKI survivors are also at risk for HZ using one of the largest databases in the world. We also demonstrated that, using the Mahalanobis metric matching method, propensity score matching can be applied to three (or more than three) groups of patients to achieve quasi-randomization for further analysis. By this method, a novel platform for outcome comparison under similar baseline comorbidities was provided. Also, we pointed out that the risk of HZ was as high in AKI survivors even with renal recovery. Therefore, it is consistent with our hypothesis that the altered immune system during AKI, instead of the worsening renal function alone, is responsible for the increased risk of HZ. These findings are noteworthy for clinicians taking care for any individual who survived dialysis-requiring AKI.

AKI can lead to a wide range of devastating short- and long-term sequels, in addition to the well-known risk of CKD development and mortality^7^,^21^,^22^. Besides fluid and waste removal, the kidney also functions as an immune-modulatory organ^23^, and AKI can cause short- and long-term immune-modulation both inside and outside the kidney. For example, not only neutrophil and CD4+ T-cell infiltration in mice kidneys last for at least 6 weeks after ischemic-reperfusion injury, the splenic T-cell interferon-gamma (IFN-c) production in the same animal also sustained at least to the same time point^24^. CD4 + T lymphocytes also display effector-memory phenotype for up to 11 weeks after I/R injury in mice^25^. Other organs like the heart and liver have prompt tissue leukocyte infiltration and local TNF-α and IL-1 expression hours to days after ischemic renal injury leading to apoptosis and inflammation^26^,^27^, a phenomenon known as “organ cross-talk^28^”. Besides, by modifying adhesion molecule expression and physical characteristics of neutrophils, the kidney could also modulate leukocyte trafficking in distant organs^23^,^29^. Protein binding uremic toxin accumulated during AKI also could lead to epigenetic modifications, producing distinct genomic signatures and potentially modulating cellular response^30^,^31^,^32^. These are all possible explanations for the increased risk of sepsis^13^, tuberculosis^12^ and HZ after AKI, irrespective of renal function change.

Our findings suggest that AKI patients should be closely followed-up even after recovery, because the risk of developing HZ in AKI patients was not significantly different between the recovery and non-recovery groups. In fact, the incidence was even higher in the AKI-recovery group versus non-recovery group (8.2 per 1,000 person-years and 6.9 per 1,000 person-years, respectively), although not reaching significant difference. It is also consistent with the accumulating evidence which suggests that the presence of underlying CKD modifies the outcomes of AKI, but does not completely account for the impact of AKI^33^. HZ vaccination is recommended by the Advisory Committee on Immunization Practices for persons aged 60 years or older, and the Food and Drug Administration approved its use to prevent herpes zoster in persons 50 years of age or older^15^. Our findings may help modifying vaccination policies in special patient groups including AKI survivors^34^.

There are several limitations of our study. First, because the administrative data have varying sensitivity for diagnosis, we cannot completely exclude misclassification at baseline. Although Waikar *et al* had validated the diagnosis accuracy of ICD-9-CM codes for AKI^35^, we want to be as precise as possible about patient selection. That is why we enrolled only the most severe form of AKI patients (i.e. dialysis dependent) due to the undisputable accuracy of the procedure code of hemodialysis/CRRT in the database. Secondly, laboratory data were not available in the LIHD database, therefore the temporal change of estimated glomerular filtration rate (eGFR) after discharge was also lacking. However, our previous single-center study on surgical intensive care unit patients showed that 29.5% patients had preserved eGFR and 38.5% of non-dialysis requiring AKI survivors had eGFR deterioration into CKD stages 3–5 at 90 days after discharge^21^. The results of the previous chart-review study and the current population-based study are consistent.

Another limitation is that the diagnosis of HZ is often made upon the clinical findings of typical skin rash and blisters alone in the dermatome without further tests or laboratory examinations^15^. However, misclassification is often moderate and the accuracy of such diagnosis is high in literature population studies^36^. In order to validate the accuracy of diagnosis coding, we also performed chart review in a single medical facility and found the high positive predictive value of 90%. Sauerbrei *et al.* demonstrated that 95% of 100 clinically diagnosed HZ cases were positive for VZV viral DNA in the base of the blisters by PCR study, indicating that the clinical and virology diagnosis are concordant in most cases^36^. Besides, McDonald *et al.* also demonstrated excellent agreement between the ICD-9-CM diagnosis of herpes zoster and diagnosis by chart review^17^. The incidence of HZ has been reported to be quite constant across the world in 19 previous population studies (median 3.2 per 1,000 person-years)^37^,^38^. The incidence of HZ in our control group (4.8 per 1,000 person-years) is slightly higher than average, as our enrollees were hospitalized patients with multiple comorbidities^19^,^20^,^39^,^40^.

## Conclusion

From a population-based perspective, we found for the first time that survivors after an episode of dialysis-requiring AKI are more likely to develop HZ than those without AKI. Furthermore, all patients with or without recovery from AKI are at the same risk of HZ reactivation, and this increased risk is irrelevant to post-discharge immunosuppressant agent use. Because HZ is a significant global health burden, our findings suggest that AKI patients should be closely followed-up after the insult for both renal and extra-renal disorders. It may be necessary to enhance the post-discharge follow-up of AKI patients, even among those who have recovered from temporary dialysis.

## Methods

### Study Population and Study Design

All study cohorts were retrieved from the Longitudinal Health Insurance Database (LHID2005) in Taiwan. The National Health Insurance (NHI) program in Taiwan covers almost 99% of the entire population of 22 million people^41^. The National Health Research Institute (NHRI) used the original data from the NHI database to construct a longitudinal database of patients who had been admitted between 1999 and 2008. This cohort included 2,619,534 hospitalized patients, representing 10% of all NHI enrollees, and included demographics, disease and procedure codes for both clinical visits and hospitalization and medical costs and payments. This sampling fraction (a 3.4:1 ratio) was based on a regulation that limits the maximal amount of NHI data that can be extracted for research purposes^42^. Pre-admission comorbidities were determined as a specific entity recorded in at least one hospital admission or at least three outpatient department visits during the year prior to the index hospitalization, which was a relatively strict criterion and was well validated with good predicting power^43^,^44^,^45^.

### Ethical considerations

Informed consent was originally obtained by the NHRI, and since patients were anonymous in the present study, informed consent was not required. Also, since the identification numbers of all individuals in the NHRI databases were encrypted to protect the privacy of the individuals, this study was exempt from a full ethical review by the institutional review board of National Taiwan University Hospital (201212021RINC)^41^.

### Identification of cases and controls

**Figure 2:**
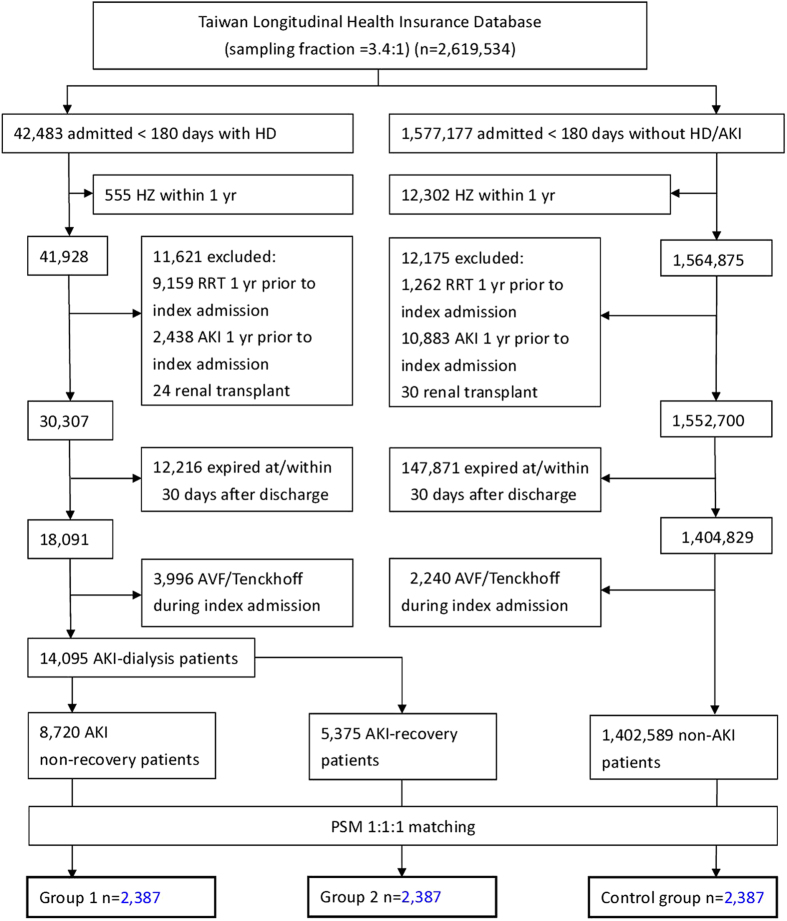
Abbreviations: *AKI* acute kidney injury, *ESRD* end-stage renal disease, HZ herpes zoster, PSM propensity score matching.

All adult patients who claimed for hemodialysis (HD) or continuous renal replacement therapy (CRRT) during an index hospitalization less than 180 days were considered eligible study subjects (N = 42,483) (Fig. 2). We did not include patients with claims of in-hospital peritoneal dialysis (PD) because AKI is seldom treated so in Taiwan. To exclude pre-existing HZ and ESRD before the index hospitalization, those who had a diagnosis of HZ (n = 555) (ICD-9-CM codes shown in supplementary table 1) or those with claims of renal replacement therapy (RRT) (n = 9,159) within 1 year prior to index admission were excluded. Likewise, to ensure that the AKI was *de novo*, those ever coded with AKI within 1 year prior to index admission (n = 2,438) were also excluded, as well as those who ever received kidney transplant (n = 24) at any time point before. 12,216 patients who died shortly after discharge were excluded. The creation of an arteriovenous shunt/graft or the insertion of a Tenckhoff catheter during the index admission suggest that rapid renal function deterioration was expected and the patient might resume renal replacement therapy shortly, so these patients were also excluded (n = 3,996). Finally, there were 8,720 patients whose renal function did not recover from dialysis-requiring AKI and went on subsequent maintenance dialysis (non-recovery group, either on HD or PD), and 5,375 patients who recovered from dialysis-requiring AKI for at least 30 days after discharge without creating dialysis access during the index hospitalization were enrolled as the study group (recovery group). On the other hand, those with neither claims of HD/CRRT nor codes of AKI during an index admission less than 180 days were regarded as the control cohort. The exclusion processes were the same as study group. The index hospitalization dates of the matched control had to be within the same year as those of the study group. The negative predictive rate of ICD-9 coding for AKI was 96.1% according to previous studies^35^,^42^, therefore the possibility of undetected AKI not requiring HD in the control group is low. We use propensity score matching in a 1:1:1 manner to achieve the comparability among the three groups for comparison. The medication prescribed after the index hospitalization was identified by the Anatomical Therapeutic Chemical (ATC) codes (Supplementary Table 1).

In order to validate the accuracy of diagnosing HZ, we performed separate chart reviewing of 110 patients with the coding of ICD-9 053.X in a single center of National Taiwan University Hospital, Hsin-Chu Branch. A hundred of the 110 patients were confirmed to have HZ reactivation by the description of typical blisters distributed over dermatomes or typical post-herpetic neuralgia, photography, or the prescription of antiviral agents. The validated positive predictive value to HZ using ICD-9 (053.X) was 90%.

### Study End Point

Each patient was followed after discharge until the development of herpes zoster, and censored at death or on December 31, 2009, whichever occurred first.

### Statistical Analysis

To reduce the selection bias caused by the differences in the baseline risks for AKI, propensity score analysis was conducted. Briefly, we conducted a multivariable logistic regression model to estimate the probability of developing dialysis-requiring AKI for every patient with demographics, index hospitalization characteristics and operative categories (if applicable) listed in Table 1. Each patient would have a probability of y1 being AKI non-recovery, y2 of being AKI recovery and y3 of being non-AKI during the index admission, and y1 + y2 + y3 equals 1, or 100%. Then, each subject was matched with subjects with the nearest Mahalanobis distance by the logit of the estimated propensity score in a 1:1:1 fashion. The chosen size of the caliper was:
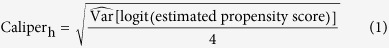


The sample variance of the logit (estimated propensity score) was 5.57, 3.61, and 5.71 for propensity scores of being AKI non-recovery, AKI recovery and non-AKI, respectively. The distribution of the logit of the estimated propensity scores before and after matching was shown in supplementary figure 1, which demonstrates clearly that the curves became identical after the matching process. Then, we went on comparing group differences before and after propensity score matching in demographic characteristics, comorbidities, and index hospitalization conditions by Kruskal-Wallis Rank sum test (for continuous variables) and Chi-square test (for categorical variables). The result showed a quasi-randomization effect of the propensity score matching, that is, the difference between the three groups became insignificant after the process.

After making sure that the groups were perfectly matched, we went on comparing the incidence of herpes zoster by Poisson regression model. Finally, we performed a Cox proportional hazards model with time-dependent covariates (post-discharge steroids and immunosuppressant agents use) to identify factors influencing HZ risk. All analyses were performed using R statistical package version 2.15.2. Two-tailed P < 0.05 was considered statistically significant.

## Additional Information

**How to cite this article**: Yang, W.-S. *et al.* High Risk of Herpes Zoster among Patients with Advance Acute Kidney Injury – A Population-Based Study. *Sci. Rep.* **5**, 13747; doi: 10.1038/srep13747 (2015).

## Supplementary Material

Supplementary Information

## Figures and Tables

**Table 1:** Baseline demographics of dialysis-dependent, dialysis-free AKI survivors and non-AKI patients before and after propensity score-matching.

Variables	*Before Matching*				*After Matching*			
	Without recovery from dialysis and AKI (Group 1) (n = 8720)	With recovery from dialysis and AKI (Group 2) (n = 5375)	Without dialysis or AKI (Group 3) (n = 1402589)	*p*#-value	Without recovery from dialysis and AKI (Group 1) (n = 2387)	With recovery from dialysis and AKI (Group 2) (n = 2387)	Without dialysis or AKI (Group 3) (n = 2387)	*p*#-value
Male, n (%)	4165 (47.8%)	2995 (55.7%)	644879 (46%)	<0.001	1278 (53.5%)	1262 (52.9%)	1298 (54.4%)	0.578
Age, mean (SD^a^), years	61.6 (14.5)	64.1 (16.27)	47.0 (18.7)	<0.001	63.0(15.6)	63.3 (15.9)	63.2 (16.5)	0.429
Co-morbidities within 1 year prior to index hospitalization, % (n)								
Charlson morbidity index, mean (SD)	3.0 (1.9)	2.2(2.1)	0.4 (1.0)	<0.001	2.0 (1.9)	2.0 (1.9)	2.1 (2.0)	0.864
Myocardial infarction, n (%)	201 (2.3%)	222 (4.1%)	7178 (0.5%)	<0.001	49 (2.1%)	40 (1.7%)	59 (2.5%)	0.154
Congestive heart failure, n (%)	1239 (14.2%)	752 (14%)	17573 (1.3%)	<0.001	255 (10.7%)	240 (10.1%)	238 (10%)	0.675
Peripheral vascular disease, n (%)	94 (1.1%)	90 (1.7%)	3874 (0.3%)	<0.001	21 (0.9%)	23 (1%)	21 (0.9%)	0.940
Cerebrovascular disease, n (%)	844 (9.7%)	609 (11.3%)	44081 (3.1%)	<0.001	217 (9.1%)	223 (9.3%)	193 (8.1%)	0.270
Dementia, n (%)	92 (1.1%)	140 (2.6%)	8747 (0.6%)	<0.001	26 (1.1%)	34 (1.4%)	21 (0.9%)	0.200
COPD^b^, n (%)	805 (9.2%)	671 (12.5%)	72001 (5.1%)	<0.001	226 (9.5%)	215 (9%)	216 (9%)	0.830
Rheumatologic disease, n (%)	130 (1.5%)	82 (1.5%)	6700 (0.5%)	<0.001	33 (1.4%)	27 (1.1%)	27 (1.1%)	0.658
Peptic Ulcer, n (%)	1214 (13.9%)	764 (14.2%)	80222 (5.7%)	<0.001	232 (9.7%)	281 (11.8%)	266 (11.1%)	0.066
Hemiplegia, n (%)	108 (1.2%)	78 (1.5%)	6204 (0.4%)	<0.001	20 (0.8%)	32 (1.3%)	28 (1.2%)	0.243
Cancer, n (%)	394 (4.5%)	369 (6.9%)	50151 (3.6%)	<0.001	133 (5.6%)	109 (4.6%)	130 (5.4%)	0.233
Diabetes Mellitus, n (%)	3810 (43.7%)	1935 (36%)	95144 (6.8%)	<0.001	828 (34.7%)	827 (34.6%)	834 (34.9%)	0.974
Moderate or Severe liver disease, n (%)	493 (5.7%)	381 (7.1%)	40560 (2.9%)	<0.001	98 (4.1%)	109 (4.6%)	134 (5.6%)	0.043
Chronic Kidney disease, n (%)	4439 (50.9%)	836 (15.6%)	3602 (0.3%)	<0.001	455 (19.1%)	427 (17.9%)	442 (18.5%)	0.579
Dyslipidemia/hyperlipidemia, n (%)	859 (9.9%)	580 (10.8%)	39400 (2.8%)	<0.001	185 (7.8%)	203 (8.5%)	192 (8%)	0.629
Hypertension, n (%)	5279 (60.5%)	2561 (47.6%)	195395 (13.9%)	<0.001	1104 (46.3%)	1146 (48%)	1156 (48.4%)	0.278
Coronary heart disease, n (%)	869 (10%)	751 (14%)	52576 (3.7%)	<0.001	225 (9.4%)	207 (8.7%)	231 (9.7%)	0.459
Obesity, n (%)	3 (0%)	5 (0.1%)	902 (0.1%)	0.386	2 (0.1%)	1 (0%)	2 (0.1%)	0.819
Alcoholism, n (%)	2 (0%)	3 (0.1%)	496 (0%)	0.601	1 (0%)	2 (0.1%)	0 (0%)	0.368
HIV^c^ infection, n (%)	2 (0%)	4 (0.1%)	610 (0%)	0.362	1 (0%)	3 (0.1%)	6 (0.3%)	0.149
Transplant, n (%)	16 (0.2%)	41 (0.8%)	1588 (0.1%)	<0.001	3 (0.1%)	6 (0.3%)	2 (0.1%)	0.306
Cause of index hospitalization								
Cardiovascular, n (%)	82 (0.9%)	410 (7.6%)	6325 (0.5%)	<0.001	29 (1.2%)	34 (1.4%)	35 (1.5%)	0.726
Respiratory, n (%)	422 (4.8%)	1077 (20%)	12400 (0.9%)	<0.001	194 (8.1%)	209 (8.8%)	204 (8.5%)	0.730
Hepatic, n (%)	69 (0.8%)	106 (2%)	7165 (0.5%)	<0.001	18 (0.8%)	15 (0.6%)	16 (0.7%)	0.866
Neurologic, n (%)	112 (1.3%)	103 (1.9%)	1822 (0.1%)	<0.001	27 (1.1%)	34 (1.4%)	32 (1.3%)	0.654
Hematologic, n (%)	55 (0.6%)	82 (1.5%)	3639 (0.3%)	<0.001	21 (0.9%)	25 (1%)	20 (0.8%)	0.725
Metabolic, n (%)	169 (1.9%)	169 (3.1%)	524 (0%)	<0.001	37 (1.6%)	43 (1.8%)	27 (1.1%)	0.156
Operative categories								
Cardiothoracic, n (%)	89 (1%)	216 (4%)	6929 (0.5%)	<0.001	28 (1.2%)	39 (1.6%)	38 (1.6%)	0.342
Upper gastrointestinal, n (%)	11 (0.1%)	42 (0.8%)	7969 (0.6%)	<0.001	6 (0.3%)	5 (0.2%)	6 (0.3%)	0.943
Lower gastrointestinal, n (%)	21 (0.2%)	106 (2%)	11456 (0.8%)	<0.001	9 (0.4%)	11 (0.5%)	15 (0.6%)	0.448
Hepato-biliary, n (%)	18 (0.2%)	64 (1.2%)	24248 (1.7%)	<0.001	9 (0.4%)	5 (0.2%)	14 (0.6%)	0.112
ICU admission during index hospitalization, n (%)	2231 (25.6%)	3594 (66.9%)	82823 (5.9%)	<0.001	1009 (42.3%)	1009 (42.3%)	998 (41.8%)	0.933
Risk factors after index hospitalization								
Steroids	4227 (48.5%)	2515 (46.8%)	550315 (39.2%)	<0.001	1079 (45.2%)	1168 (48.9%)	985 (41.3%)	<0.001
Hydroxychloroquine	88 (1%)	68 (1.3%)	11702 (0.8%)	0.001	28 (1.2%)	35 (1.5%)	25 (1%)	0.403
Azathioprine	48 (0.6%)	60 (1.1%)	2929 (0.2%)	<0.001	15 (0.6%)	28 (1.2%)	6 (0.3%)	0.001
Mycofenolate mofetil	318 (3.6%)	72 (1.3%)	574 (0%)	<0.001	71 (3%)	41 (1.7%)	3 (0.1%)	<0.001
Cyclosporine	208 (2.4%)	56 (1%)	1625 (0.1%)	<0.001	47 (2%)	30 (1.3%)	2 (0.1%)	<0.001
Cyclophosphamide	54 (0.6%)	59 (1.1%)	10721 (0.8%)	0.006	20 (0.8%)	31 (1.3%)	21 (0.9%)	0.211
Outcome								
Herpes Zoster, n (%)	245 (2.8%)	121 (2.3%)	18284 (1.3%)	<0.001	56 (2.3%)	65 (2.7%)	42 (1.8%)	0.080

^#^*p* value refers to Kruskal–Wallis test for continuous variables and Chi-square test for categorical variables (non-recovery group vs. recovery group vs. non-AKI group).

^a^SD, standard deviation.

^b^COPD, chronic obstructive pulmonary disease.

^c^HIV, human immunodeficiency virus.

**Table 2:** Cox regression analysis of 2,387 matched trios^a^ for identifying the risk factors for herpes zoster reactivation with time-varying covariates.

Covariate	Hazard ratio	Lower 95% CI	Upper 95% CI	*p*-value
Logit of estimated propensity score 1 (> or ≦0.388)^b^	1.65	0.94	2.92	0.082
Logit of estimated propensity score 2 (< or ≧−6.404)^c^	1.10	0.76	1.60	0.606
Reversed age^d^	1.08	1.04	1.12	<0.001
Actual age^e^	1.02	1.01	1.04	0.002
Moderate to severe liver disease^f^	0.34	0.08	1.36	0.127
Transplant^g^	6.16	1.46	25.96	0.013
Immunosuppressive drug used after index discharge				
Steroids	2.51	1.54	4.09	<0.001
Mycofenolate mofetil	8.22	3.74	18.05	<0.001
Hydroxychloroquine	9.71	4.11	22.94	< 0.001
Cyclosporine	2.52	1.01	6.26	0.047
Non-AKI^h^ patients vs AKI patients	0.65	0.45	0.94	0.021

^#^concordance = 0.69.

^a^The 2,387 trios of subjects were matched by the logit (estimated propensity score), thus the logits of the estimated propensity scores was purposefully kept in the regression model to reduce selection bias.

^b^Due to the non-linearity distribution of the logit of estimated propensity score 1, a cut-off value was set at 0.388 and this covariate is transferred from a continuous into a categorical variable.

^c^Due to the non-linearity distribution of the logit of estimated propensity score 2, a cut-off value was set at −6.404 and this covariate is transferred from a continuous into a categorical variable.

^d^The risk of HZ was not linear to patient’s age in the generalized additive model (GAM) plot (supplement figure 2). Therefore, age was presented in a reverse way ( i.e. the subtraction of the actual age from the eldest age among all patients, that is, 96.8 years-old) for patients older than the turning point, that is, 70.4 years-old.

^e^For patients younger than 70.4 years old, same reason as mentioned above.

^f^Due to the fact that there was slightly more patients with moderate to severe liver disease in non-AKI group, this variable is kept in the model purposefully to avoid selection bias.

^g^Solid organ (except kidney) or hematopoietic transplantation.

^h^AKI, acute kidney injury.
