# Supplementary material for: High Risk of Herpes Zoster among Patients with Advance Acute Kidney Injury – A Population-Based Study
Source: Sci Rep. 2015 Sep 3;5:13747. doi: 10.1038/srep13747 (PMC4558719; doi:10.1038/srep13747)
Supplement: Supplementary Information [file srep13747-s1.doc]

**High Risk of Herpes Zoster among Patients with Advance Acute Kidney Injury – A Population-Based Study**

Wei-Shun Yang^1,2^, Fu-Cheng Hu^3^, Meng-Kan Chen^5^, Wen-Je Ko^6^, Likwang Chen^7^, Kwan-Dun Wu ^2,4^, Vin-Cent Wu^2,4^

^1^ Department of Internal Medicine, National Taiwan University Hospital, Hsin-Chu Branch, Hsin-Chu city, Taiwan

^2^ College of Medicine, National Taiwan University, Taipei, Taiwan

^3^ International Harvard Statistical Consulting Company, Taipei, Taiwan

^4^ Division of Nephrology, Department of Internal Medicine, National Taiwan University Hospital, Taipei, Taiwan

^5^ Department of Family Medicine, National Taiwan University Hospital, Hsin-Chu Branch, Hsin-Chu, Taiwan

^6^ Department of Surgery, National Taiwan University Hospital, Taipei, Taiwan

^7^ Institute of Population Health Sciences, National Health Research Institutes, Zhunan, Taiwan

Corresponding author: Vin-Cent Wu

Department of Internal Medicine, National Taiwan University Hospital, 7 Chung-Shan South Road, Taipei, 10002, Taiwan

Telephone: +886-2-2356 3549

Fax: +886-2-23819383

E-mail: [q91421028@ntu.edu.tw](mailto:q91421028@ntu.edu.tw)

**Supplementary Table 1. International classification of disease (ICD) 9 code used in the present study**

1. Comorbidity

|  | **ICD9-codes** |
| --- | --- |
| **Myocardial infarction** | 410, 410.X, 410.XX, 412 |
| **Congestive heart failure** | 428,428.X |
| **Peripheral vascular disease** | 441, 441.X, 443.9, 785.4, V434 |
| **Cerebrovascular disease** | 430, 431, 432, 432.X, 433, 433.X, 434, 434.X, 435, 435.X, 436, 437,437.X, 438,438.X, 438.XX |
| **Dementia** | 290, 290.X, 290.XX |
| **Chronic pulmonary disease** | 490, 491, 491.X, 491.XX,492, 492.X, 493, 493.X, 493.XX,494, 495, 495.X, 496, 500, 501, 502, 503, 504, 505, 506.4 |
| **Rheumatologic disease** | 710.X, 714, 714.X, 714.XX, 725 |
| **Peptic Ulcer** | 531, 531.X, 531.XX,532, 532.X, 532.XX,533, 533.X, 533.XX,534, 534.X, 534.XX |
| **Moderate or Severe liver disease** | 571.2, 571.4, 571.4X, 571.5, 571.6, 572, 572.X, 456, 456.0, 456.1, 456.2, 456.2X |
| **Diabetes Mellitus** | 250, 250.0, 250.0X, 250.1, 250.1X, 250.2, 250.2X, 250.3, 250.3X, 250.4, 250.4X, 250.5, 250.5X, 250.6, 250.6X, 250.7, 250.7X |
| **Hemiplegia** | 342, 342.X, 342.XX, 344.1 |
| **Cancer** | 140,140.X,141, 141.X,142, 142.X,143, 143.X,144, 144.X,145, 145.X,146,146.X,147,147.X,148,148.X,149,149.X,150,150.X  , 151, 151.X, 152, 152.X, 153, 153.X, 154, 154.X, 155, 155.X  , 156, 156.X, 157, 157.X, 158, 158.X, 159, 159.X,160,160.X, 161, 161.X, 162, 162.X, 163, 163.X, 164, 164.X, 165, 165.X, 166  , 166.X, 167, 167.X, 168, 168.X, 169, 169.X, 170,170.X,171,171.X,172,172.X, 174,174.X,175,175.X,176,176.X,179,180,180.X,181,182,182.X  ,183,183.X,184,184.X,185,186,186.X,187,187.X,188,188.X,189  ,189.X, 190,190.X,191,191.X,192,192.X,193,194,194.X,195  ,195.X, 200,200.X,200.XX,201,201.X, 201.XX,202,202.X,202.XX,  203,203.X,203.XX,204,204.X,204.XX,205,205.X,205.XX,206  ,206.X,206.XX,207,207.X,207.XX,208,208.X,208.XX |
|  | 196,196.X,197,197.X,198,198.X,198.XX,199,199.X, |
| **Chronic Kidney disease** | 585 |
| Hypertension | 401, 401.X, 401.XX, 402, 402.X, 402.XX, 403, 403.X, 403.XX, 404, 404.X, 404.XX, 405, 405.X, 405.XX, |
| **Hyperlipidemia** | 272,2720,2721,2722,2723,2724,2725,2726,2727,2728,2729 |
| **Coronary heart disease** | 410,4100,41000,41001,41002,4101,41010,41011,41012,4102,41020,41021,41022,4103,41030,41031,41032,4104,41040,41041,41042,4105,41050,41051,41052,4106,41060,41061,41062,4107,41070,41071,41072,4108,41080,41081,41082,4109,41090,41091,41092,411,4110,4111,4118,41181,41189,412,413,4130,4131,4139,414,4140,41400,41401,41402,41403,41404,41405,4141,41410,41411,41419,4148,4149,4292 |
| **Obesity** | 278,2780,27800,27801 |
| **Alcohol** | 303,3050 |
| **HIV infection** | 042-044, V08 |
| **Transplant** | 00.91-93, 33.5, 37.51, 33.6, 41.0X, 46.97, 50.5X, 52.8X, 55.6 |

1. Acute organ dysfunction

|  | **ICD9-codes** |
| --- | --- |
| **Cardiovascular** | 458, 458.9, 785.5, 785.51, 785.59, 796.3 |
| **Respiratory** | 518.81, 518.82, 518.85, 786.09, 799.1, 967, 967.1, 967.2, 960.4,939 |
| **Hepatic** | 570, 572.2, 573.3, 573.4 |
| **Neurologic** | 293, 348.1, 348.3, 780.01, 780.09, 891.4 |
| **Hematologic** | 286.2, 286.6, 286.9, 287.3, 287.4, 287.5, 790.92 |
| **Metabolic** | 276.2 |

1. Dialysis and Acute kidney failure

|  | **ICD9-codes** | **Procedure code** |
| --- | --- | --- |
| **Dialysis** |  | 58001C,58019C,58020C,58021C  ,58022C,58023C,58024C,58025C  ,58027C,58029C,58007C,58014C  ,58018C,58002C,58011A,58011B  ,58011C,58017A,58017B,58017C  ,58026C,58028C |
| **AKI** | 584.X,634.3,635.3,636.3,  ,637.3,638.3,639.3,669.3,958.5 |  |

1. Major operation

| **Operative categories** | **Procedure code*** |
| --- | --- |
| **Cardiothoracic surgery** | 68001B, 68003B, 68005B, 68006B, 68007B, 68008B, 68009B, 68010B, 68011B, 68015B, 68016B, 68017B, 68018B, 68019B, 68020B, 68021B, 68022B, 68023B, 68024B, 68025B, 68026B, 68027B, 68028B, 68029B, 68032B, 68033B, 68034B, 68035B, 68036B, 68037B, 68038B, 68039B, 68046B, 68047B, 68049B, 68050B, 68051B, 68052B,67005B ,67010B, 67011B, 67013B, 67014B, 67015B, 67016B, 67019B, 67020B, 67022B, 67023B, 67024B, 67026B, 67027B, 67028B, 67029B, 67031B, 67032B, 67033B, 67036B, 67038B, 67039B, 67040B, 67042B, 67043B, 67044B, 67045B, 67047B, 67048B, 67049B, 67050B, 67051B,  69004B |
| **Upper GI** | 71201B, 71202B, 71204B, 71205B, 71206B, 71209B, 71210B, 71211B, 71213B, 71214B,  71215B, 71216B, 71217B, 71218B, 71219B,  71220B, 71221B, 71222B, 71223B, 71224B,  71225B, 72006B, 72007B, 72008B, 72009B, 72010B, 72011B, 72014B, 72015B, 72016B, 72018B, 72020B, 72026B, 72027B, 72028B, 72030B, 72031B, 72032B, 72033B, 72034B, 72035B, 72036B, 72037B, 72038B, 72039B, 72040B, 72041B, 72042B, 72043B, 72044B, 72046B, 72047B, 72048B, 72049B, |
| **Lower GI** | 73001B, 73002B, 73003B, 73006B, 73007B, 73010B ,73011B, 73012B, 73013B, 73014B, 73015B, 73017B, 73023B, 73024B, 73025B, 73027B, 73028B, 73029B, 73031B, 73038B, 73039B, 73040B, 73043B, 73045B, 73046B,  74203C, 74205B, 74206B, 74208B, 74209B, 74211B, 74213B, 74214B, 74215B, 74216B, 74217B, 74219B, 74220B, 74222B, 97420B, 97421B, 97422B, |
| **Hepatobiliary** | 75002B, 75003B, 75004B, 75005B, 75007B, 75008B, 75009B, 75010B, 75011B, 75012B, 75014B, 75015B, 75016B, 75017B, 75018B, 75019B, 75020B, 75021B, 75022B, 75023B,    75202B, 75203B, 75204B, 75205B, 75206B, 75208B, 75209B, 75210B, 75212B, 75213B, 75214B, 75215B, 75216B, 75218B,  75403B, 75404B, 75405B, 75406B,75407B, 75408B, 75409B, 75410B, 75411B, 75412B, 75413B, 75414B, 75415B, 75416B, 75417B, 75418B, 75419B, |

1. ICU

|  | **ICD9-codes** | **Procedure code** |
| --- | --- | --- |
| **ICU** |  | 03010E ,03011F ,03012G, 3047E,03048F, 03049G, 02011K,02012A ,02013B |

1. **A-V fistula and** Tenckhoff

|  | **ICD9-codes** | **Procedure code** |
| --- | --- | --- |
| **A-V fistula** |  | 69032C , 69034C |
| **Tenckhoff** |  | 58012B |

1. **Immunosuppressant** Agents

|  | **ACT codes** |  | |
| --- | --- | --- | --- |
| **Hydroxychloroquine** | P01BA02 | |  |
| **Cyclophosphamide** | L01AA01 | |  |
| **Azathioprine** | L04AX01 | |  |
| **Cyclosporine** | L04AD01, S01XA18 | |  |
| **Mycofenolate mofetil** | L04AA06 | |  |
| **Steroid** | H02A, H02B | |  |

1. Outcome

|  | **ICD9-codes** | **Procedure code** | |
| --- | --- | --- | --- |
| **Herpes zoster** | 053,0530,0531,05310,05311,05312,05313,05319,0532,  05320,05321,05322,05329,0537,05371,05379,0538,  0539 | |  |

**Supplementary Figure 1: The distribution of the logit of the estimated propensity scores before and after matching adults of AKI without or with recovery from dialysis and non-AKI patients.**

1. Logit of the estimated propensity score for being AKI non-recovery:


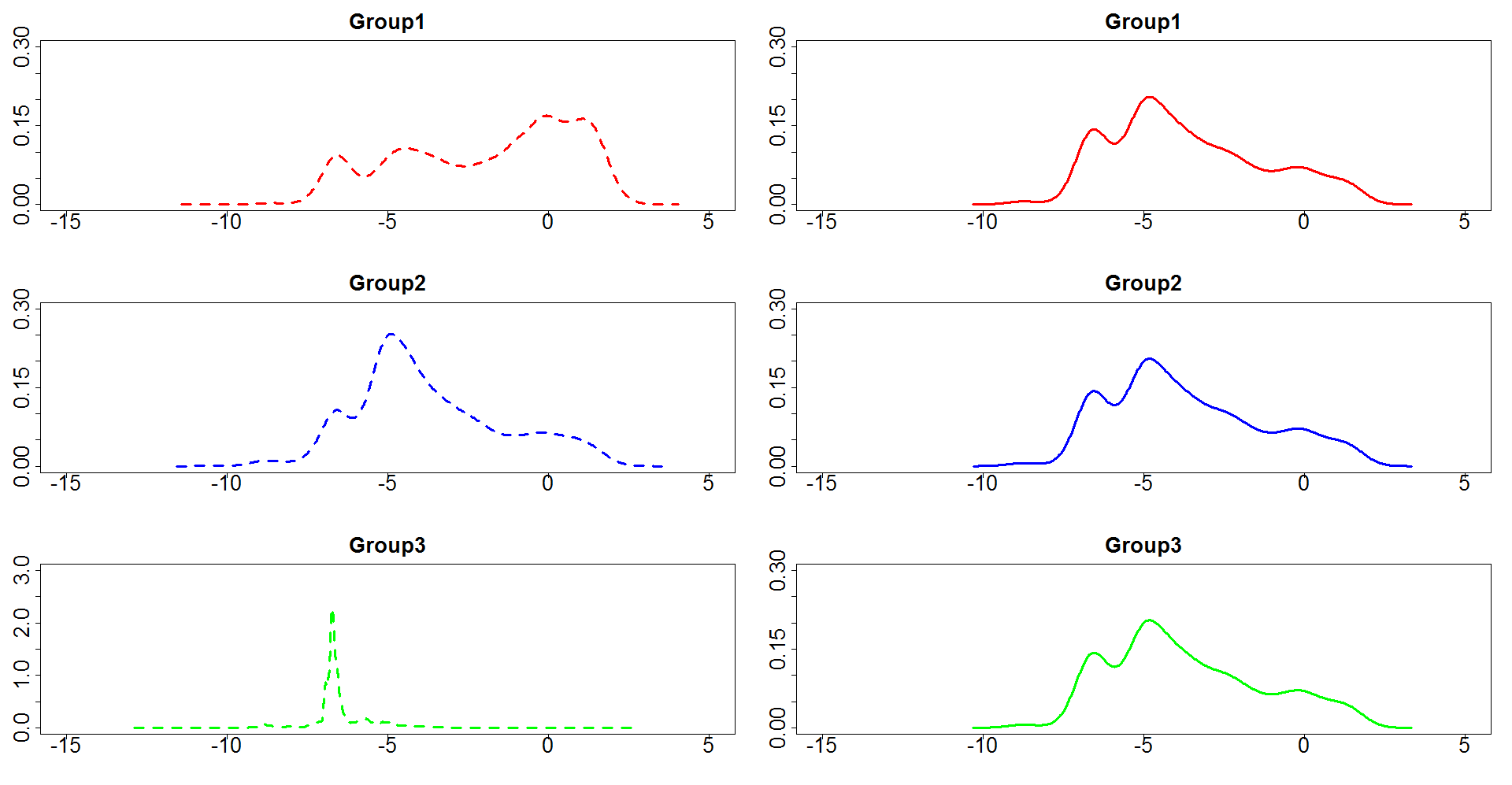


1. Logit of the estimated propensity score for being AKI recovery:


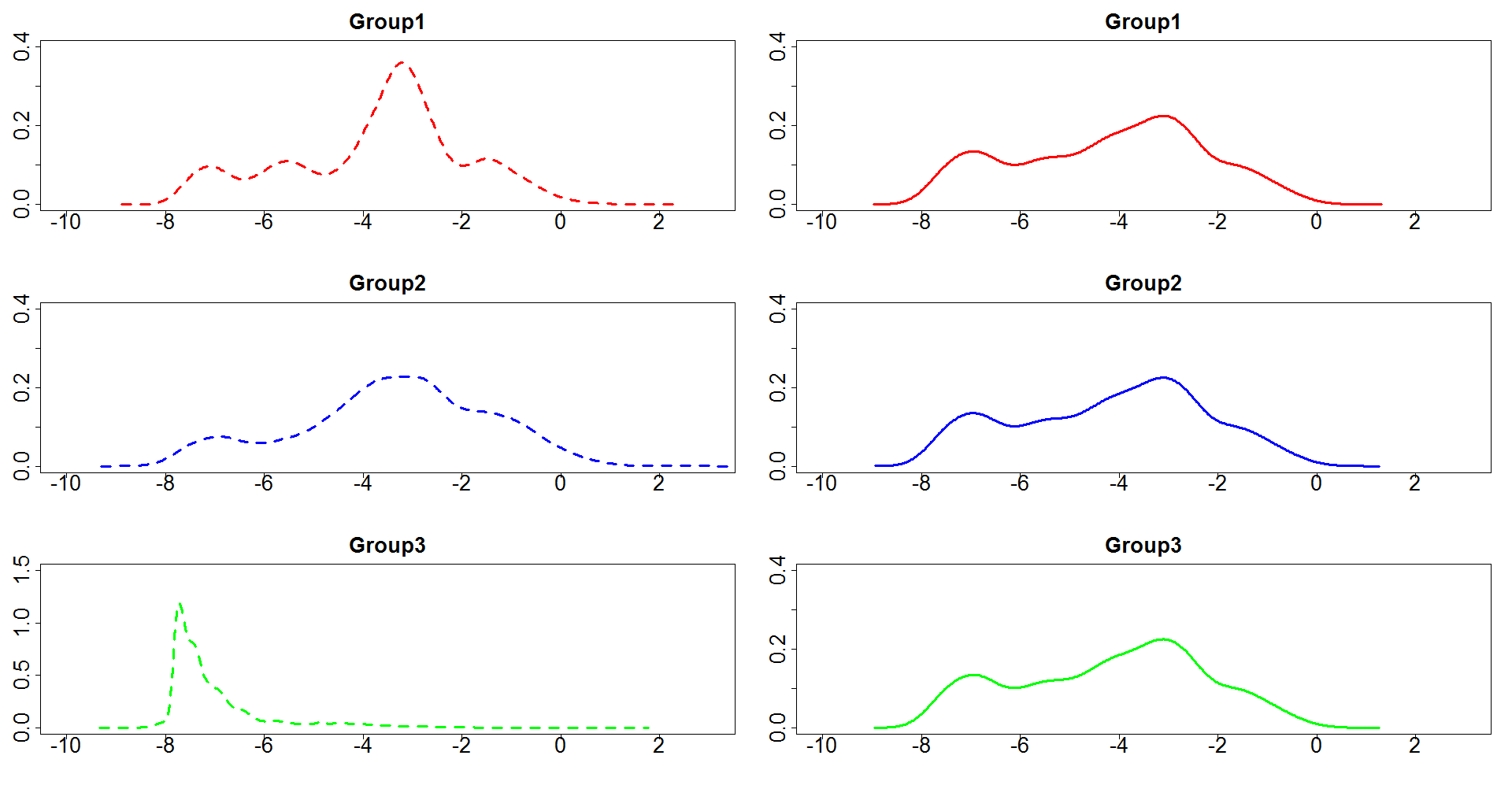


1. Logit of the estimated propensity score for being non-AKI:


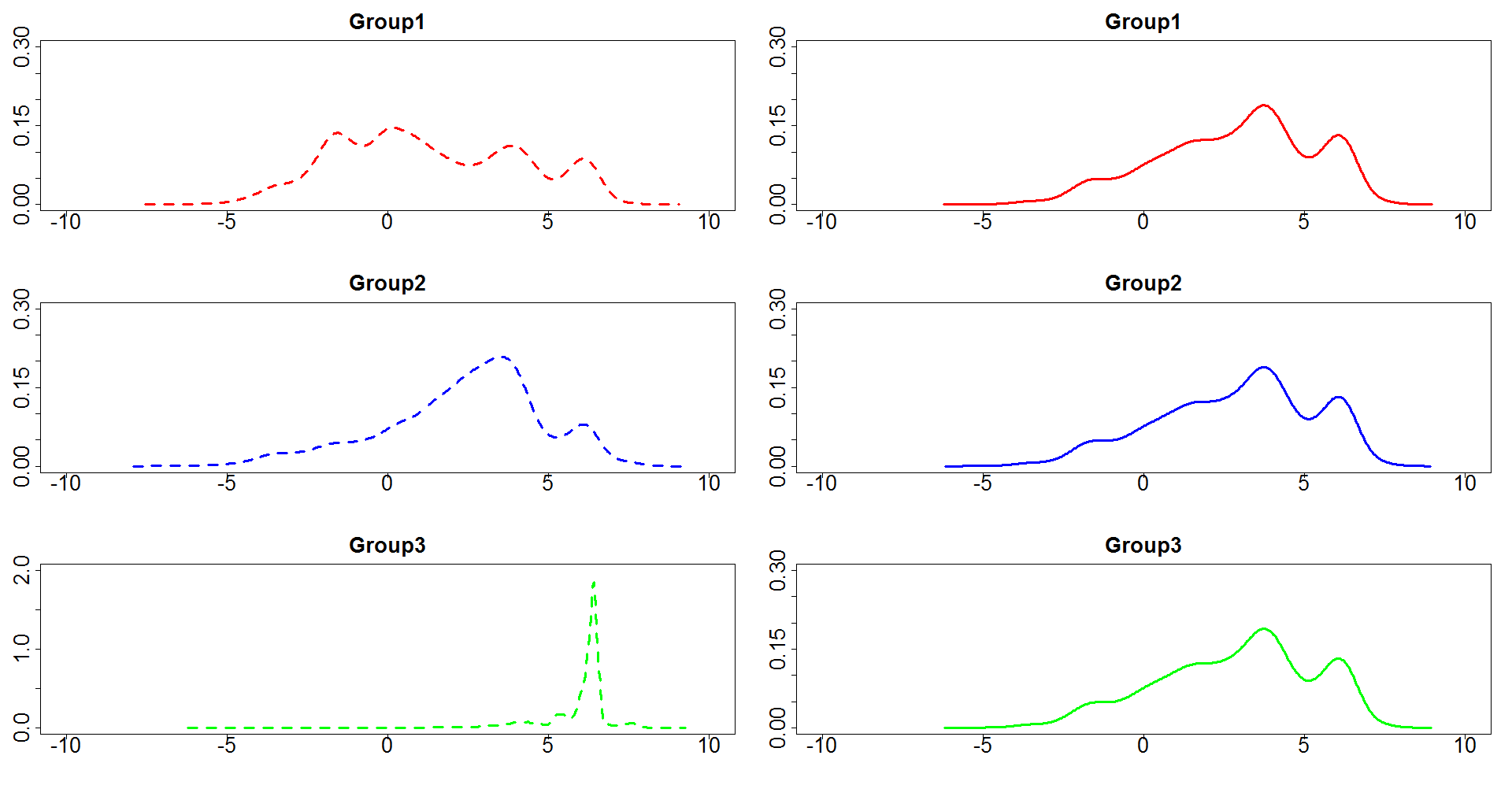


Right panel: before matching.

Left panel: after matching.

Note that after matching, the distribution of the logit became more unified for all three groups, which means the probability of a certain individual of being in AKI non-recovery, AKI recovery or non-AKI group is nearly identical with his/her paired control cases after the matching process.

**Supplementary Figure 2: generalized additive model (GAM) plot for herpes zoster event versus age**

**
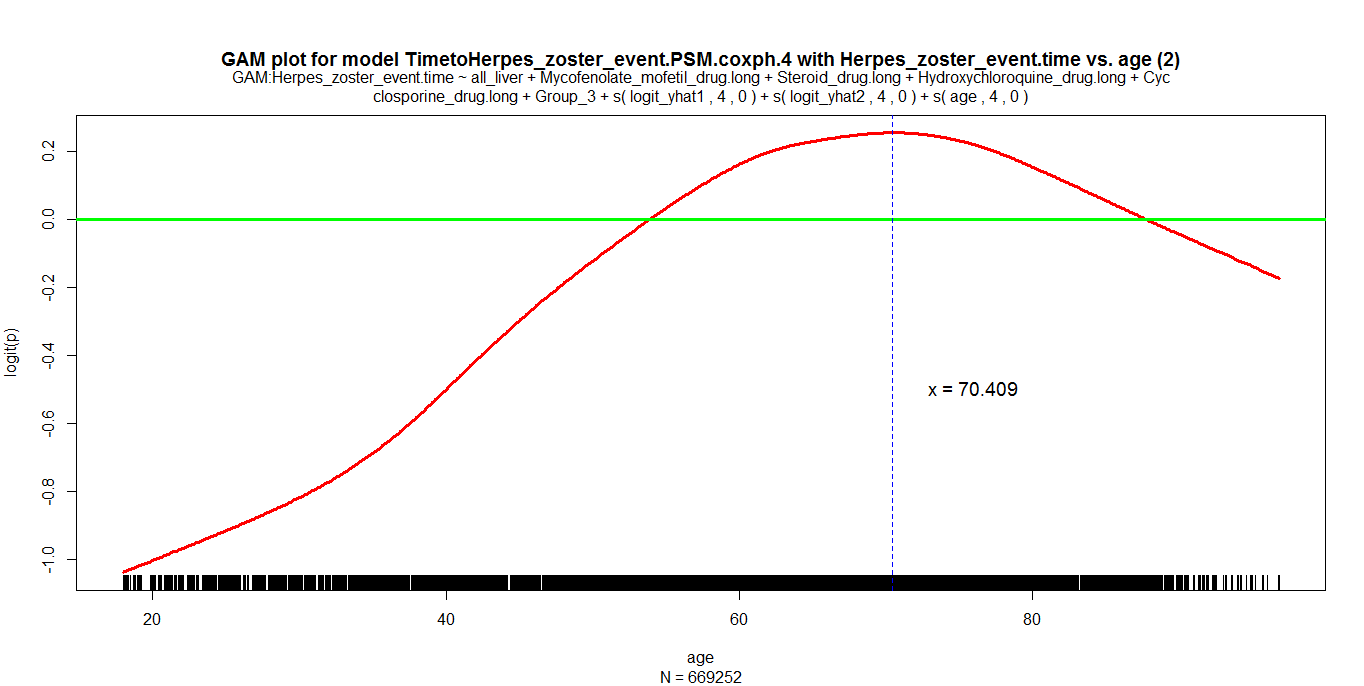
**
